# Exploratory laparoscopy – diagnosis method in pediatric surgery pathology

**Published:** 2009

**Authors:** Isabela Drăghici, Liviu Drăghici, Maria Popescu, Mircea Liţescu

**Affiliations:** * Department of Pediatric Surgery, “Maria Sklodowska Curie” Hospital, Bucharest; ** Department of General Surgery, “Sf. Ioan” Hospital, Bucharest National Center of Laparoscopic Training

**Keywords:** exploratory laparoscopy, pediatric pathology, decisional algorithms

## Abstract

The purpose of this article is to establish the important place of laparoscopy in the diagnosis management of pediatric surgery pathology. In addition, it is intended to become a pleading, concerning the true and realistic benefit, for the pediatric patient, of minimally invasive surgery.

In our country, the method was taken over from general surgery and had a few years of latency in imposing itself. We consider that each pediatric surgeon is required to have a mandatory period of training in this topic. This is the reason why we appeal to the young generations of pediatric surgeons but also to the surgeons with many years of experience in classical surgical techniques, who are able to perfect the diagnostic and treatment methods in many medical specialties.

The clinical research took place in a retro and prospective manner from August 1999 to July 2007 in the Department of Pediatric Surgery “Maria Sklodowska Curie” Hospital and studied 663 laparoscopic surgeries. 83 of them were exploratory laparoscopies (39 boys and 44 girls) and the other 580 were therapeutic procedures with accurate pre-operatory diagnostics.

The surgical activity of our department concerning exploratory laparoscopy was also appreciated due to the decisional and therapeutic algorithms that were conceived.

The motivations for exploratory laparoscopic procedures are multiple and arise from many causes, from right lower quadrant pain syndrome and abdominal tumors to congenital malformations and non-palpable testis.

The processed data revealed the utility of laparoscopy, concerning most of the pediatric surgical pathologies and in the same time allowing them to continue the exploratory method of diagnosis with a minimally invasive therapy. The discrepancy between the exploratory benefit (sometimes minor) and the surgical trauma of an open exploratory surgery, explains the utility of laparoscopy that took over most of open surgery indications.

## Introduction

In 1973, Steven Gans performed the first pediatric laparoscopy in the world, for exploratory reasons. Laparoscopic pediatric instruments were designed in 1970, when the first mini telescope was created. Klimcovich and his co-workers reported the first therapeutic thoracic minimal invasive procedure in children with benign pulmonary pathology in 1971.

In our country, the first pediatric laparoscopies were performed in August 1999 in the Department of Pediatric Surgery of “Maria Sklodowska Curie” Hospital. Under the care of “Sf. Ioan” National Laparoscopic Training Center Bucharest, a team of pediatric surgeons from our center performed the first celioscopic appendectomy. Naturally, starting with the year 2000, the video endoscopic techniques imposed themselves in pediatric surgery. This was the year the first laparoscopic diagnostic procedures in pediatric patients were performed in our country. 

The children’s age does not limit the opportunity of laparoscopic surgery, nowadays instruments with the diameter of 1,7mm allow laparoscopic procedures in newly born patients. The use of laparoscopy, as an exploratory procedure in pediatric patients, is highly recommended and sometimes irreplaceable, some surgical interventions such as laparoscopic cholecystectomy presents important advantages against conventional open surgery.

## Material and method 

The clinical research took place in a retro and prospective manner, from August 1999 to July 2007 in the Department of Pediatric Surgery “Maria Sklodowska Curie” Hospital, and studied out of 43530 surgical procedures, 663 laparoscopic surgeries. All patients with laparoscopic procedures for exploratory purpose only and procedures that went forward for surgical treatment due to different pathologies (83 cases) were selected. If the exploratory procedure was followed by a laparoscopic treatment, the case was taken into consideration in the study. Patients were followed-up after the operations until 2007.

The study did not take into consideration patients with laparoscopic procedures that did not have an exploratory purpose in the beginning and represent just a convenient surgical treatment (580 procedures). We have taken into consideration some aspects of the patients with exploratory laparoscopies, such as sex (39 boys and 44 girls) and age (2 days youngest). All data was processed statistically by a computer with the help of EPIINFO program, based on the “diagnosis intention”. 

## Results

In order to prove the importance of the exploratory laparoscopy procedures in our department we have conceived a decisional algorithm of surgical management (**Fig. 1**) and a therapeutic algorithm (**Fig. 2**) concerning the pediatric patient.

**Table no. 1** reflects the laparoscopic activity of the Department of Pediatric Surgery of “M.S. Curie” Hospital, in reference to the total number of patients admitted in that period (52779).

Waldschmidt and Schier published their 13 years of experience (1978-1991) in the domain, reporting 136 curative laparoscopies and 79 for diagnosis (Obstructive jaundice 41, ascites 6, intersex 6, and non-palpable testis 26) [**2**]. 

**Table I:** 

*Year*	* Number of patients admitted *	*Total laparoscopies (related to admittances) *	**	*Total exploratory laparoscopies (related to the total of laparoscopies) *	**
		Number	%	Number	%
1999*	3340	11	0,33	0	0
2000	6025	40	0,66	3	7,5
2001	6409	47	0,73	5	10,64
2002	6821	72	1,10	10	13,89
2003	6623	91	1,37	14	15,38
2004	6844	122	1,78	10	8,20
2005	6758	121	1,79	14	11,57
2006	6538	109	1,67	18	16,51
2007**	3421	50	1,46	9	18,00
Total	52779	663		83	
*August – December, **January – July					

Although only 2% of the procedures performed in our department are laparoscopic ones, this percent defines only partially the importance of exploratory laparoscopy. Only 75% of the patients that are admitted in our department undergo a surgical procedure and only 70% of these operations can be performed laparoscopically.

**Fig. 1:**
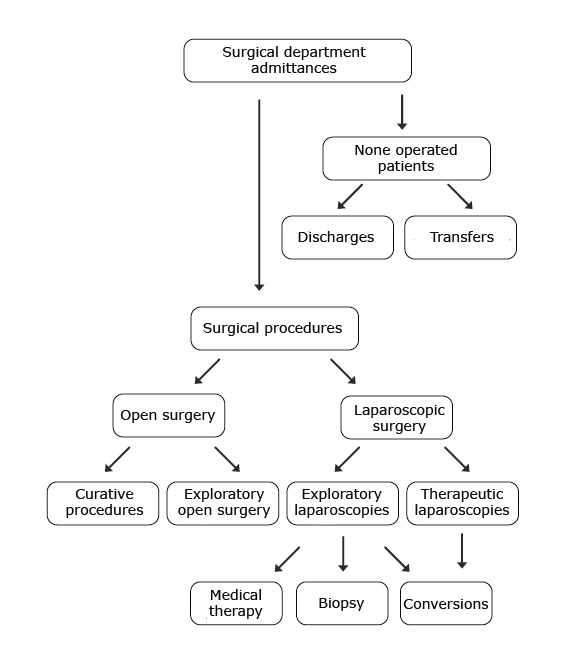
The decisional algorithm for surgical management concerning the pediatric patient

**Fig. 2:**
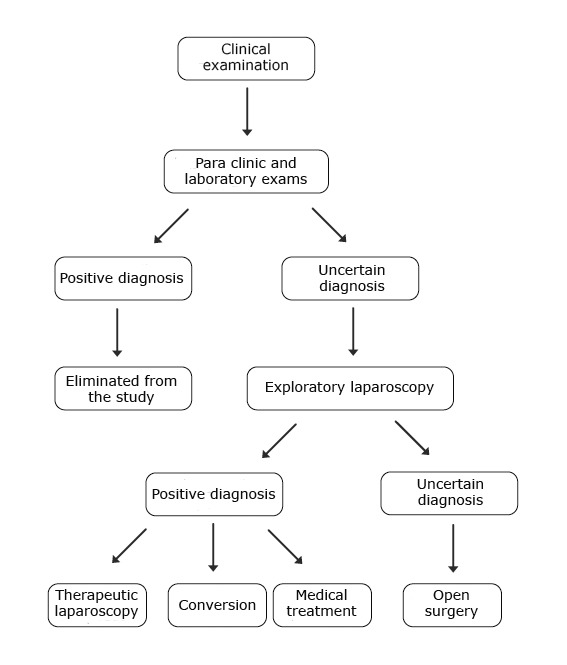
The exploratory laparoscopic algorithm concerning a pediatric patient

The development of the total number of laparoscopic procedures, during the period of study (1999-2007), describes an ascending slope with a minimum regression in 2006, probably due to technical problems that sometimes take a long time to adjust (**Diagram I**).

In time, exploratory laparoscopy became more frequently used for diagnosis. In 2007, the exploratory procedures reached 18% of the total laparoscopies, while the celioscopic procedures in general soared upward. 

Please, notice the inferior average age of children subjected to exploratory laparoscopies that in 2000 had a minimum of age, 2.2 years (**Diagram II**). Although, the youngest of our patients were 2 newly born, different sexes, 2 days old (*male diagnosed with alimentary tract malformation-intestinal obstruction, terminal ileum atresia, intestinal volvulus with rotation, intestinal duplication) and 12 days old (female diagnosed with genitourinary sinus, high vaginal atresia, hydrocolpos, meconium peritonitis, bilateral hydronephrotic urethras*).

**Diagram I:**
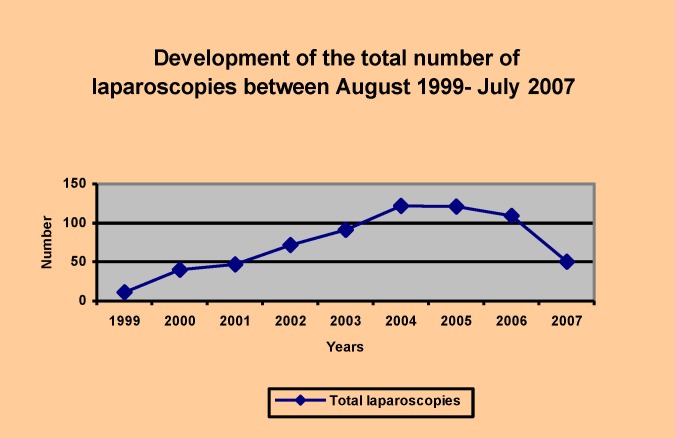


**Diagram II:**
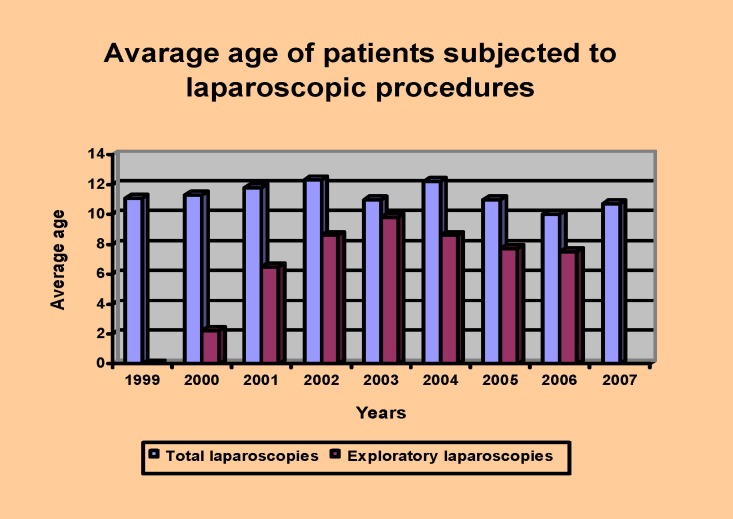


We observed a series of secondary characteristics of the cases subjected to the study that define the patient’s biological pattern.

**Table II** reflects the secondary characteristics, previously known, or newly identified during the laparoscopic exploration. 

Some of these characteristics were subjected to adjuvant medical treatment and some were not. Respiratory diseases and urinary tract infections associated to surgical pathology became the responsible factors. 

**Table II:** Secondary characteristics of studied groups

*Associated pathology*	*Number of cases*	*%*	*Associated therapy*
Respiratory pathology	4	4,81	antibiotics
Digestive pathology	3	3,61	Protons pump inhibitors , antibiotics, prokinetics
Urinary diseases	5	6,02	Urinary antiseptics
Immunodeficiency	2	2,40	Immunological modulation
Hematological disease	1	1,20	cytostatic, corticosteroids
Diabetes	1	1,20	insulin
Total	16	19,24	

The decreasing frequency of cases of hematological diseases and diabetes, in patients subjected to exploratory laparoscopies, is explained by the preference of some surgeons and anesthesiologists in our department to treat these category of patients by open surgery. 

**Table III:** 

*Procedure*	*Number of procedures ( %) *	*Conversions *	*Finalized laparoscopic procedures *
Therapeutic laparoscopies	580 (87, 5%)	29 (5%)	551 (95%)
Exploratory laparoscopies	83 (12, 5%)	6 (7,2% )	77 (92, 7%)
Total laparoscopic procedures	663 (100%)	35 (5, 2%)	628 (94, 7%)

For the study period (August 1999- July 2007) a number of 663 laparoscopic surgeries were performed, of which 580 were therapeutic procedures for accurate pre operatory diagnosis and 83 (12,5%) minimally invasive explorations. Some of the exploratory maneuvers finalized in therapeutic laparoscopic procedures or open surgery (“classical”) (**Table III**).

Montupet and Esposito [**3**] do not report conversions or complications on a group of 463 patients subjected to exploratory celioscopy, for congenital defects in the anterior abdominal wall.

**Table IV** reproduces different types of pathology, subject to exploratory laparoscopy, and it specifies intra operatory diagnosis.

**Table IV:** Pediatric pathology laparoscopic explored in our department

*Explored pathology*	*Number of cases*	*% of total exploratory laparoscopies (83)*
Right lower quadrant pain syndrome	19	22,89%
Non palpable testis	14	16,86%
Abdominal tumors	29	34,99%
Surgical emergencies	12	14,45%
Congenital malformations	9	10,84%
TOTAL	83	100%

From the total exploratory celioscopies, 19 procedures (22, 90%) were performed for *right lower quadrant pain syndrome* (acute appendicitis, ovarian cyst, Meckel diverticulitis, mesenteric lymphadenitis, genital inflammations). A special place is reserved to the most common cause in which we use exploratory celioscopy, tumor pathology (29 cases), even if the therapeutic approach does not include laparoscopy.

Ardela Diaz, Diez Pascual and Dominguez Vallejo (Department of Pediatric Surgery Burgos) recommend exploratory laparoscopy for intra operatory cholangiography, intersex diagnosis and inguinal contra lateral hernia [**4**].

## Discussion

The necessity for a new method of exploration arose from the need of a high standard diagnosis that tries to avoid unnecessary surgical procedures, sometimes dangerous in pediatric patients.

Exploratory laparoscopy emerged after a long journey in pediatric surgery. Steven Gans first named it peritonealscopy and, he first published his experience in 1973, in an attempt to gain support for the procedure [**5**] – see **Fig. 1**. 

**Fig. 1:**
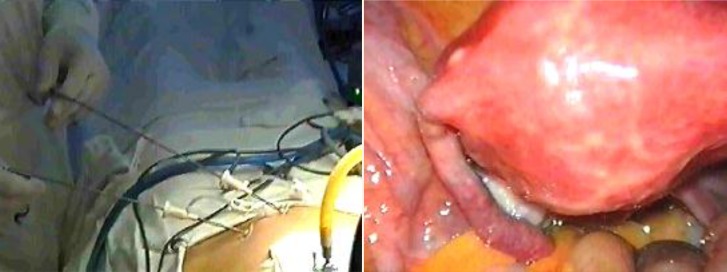
Pelvic mini exploratory laparoscopy – instruments of 2 and 3 mm

**Table:** Pediatric laparoscopic indications

		Indications and type of pathology approached laparoscopically		
Exploratory	Peritoneal	Digestive	Genital	Urologic
*Non palpable testis *	Bands and adherences	Appendectomy	Gonadectomy	Nephrectomy
*Intersex*	Appendix epiploica	Cholecystectomy	Ovarian cyst	Varicocele
*Chronic abdominal pain*	Omphalomesenteric duct	Meckel’s diverticulitis	Ectopic testis	Pyelo-uretheral stenosis
*Cholangiography*	Peritoneal dialysis catheter	Intestinal mal-rotation	Tubo-ovarian cyst	Vesico-ureteral implantation
*Biopsy*	Abscess drain	Hirschprung disease		Lymphadenectomy
*Contro lateral inguinal hernia*	Ventriculo- peritoneal catheter	Hydatid cyst		Uraca cyst
*Abdominal tumor*	Mesenteric cyst	Splenectomy		Lymphocyst
*Cancer staging *		Adrenalectomy		Renal cyst
**		Diaphragmatic hernia		

The most important indications, for exploratory celioscopic method with high efficiency, are: non palpable testis (important procedure and decisive for the future therapeutic conduit), intersex cases (indispensable, it confirms the presence or absence of the gonads and internal genital organs and it is a good method for ablation or biopsy), and also for feminizing testis [**6**, **7**].

Steven Gans attribute to exploratory laparoscopy some important indications with high profitableness (non-palpable testis, intersex) [**5**-**7**]. Olivares and Reinberg, also sustain its important utility in the study of recurring abdominal pain that has no specific cause, especially at puberty females. Laparoscopic exploration permits the ovary inspection, the detection of congenital bands, Meckel’s diverticulitis and urac cyst [**8**, **9**].

In patients with high suspicion of bile duct atresia, it is possible to perform a choleystcolangiography and a hepatic biopsy, with important value for the study and prognosis of this disease [**1**, **10**].

In abdominal trauma with penetration, hemodynamically stable, laparoscopy allows the abdominal inspection, the identification of intestinal wall lesions, diaphragm and internal organs lesions. In most of the cases, laparoscopy is an important method of treatment [**1**].

Some articles, which dwell upon laparoscopy as method of exploration, present different criteria for case selection. Usually, the most frequently used are multiple causes of abdominal pain (appendicitis, adherence syndromes, tumors, genital pathology) but also an important variety of congenital malformations.

Because the primary purpose of our study was the evaluation of the benefit of minimally invasive procedures in the exploration of the pediatric surgery pathology, we decided upon the two major criteria presented, the only ones capable to plead for the feasibility of the laparoscopic technique. Trying to limit the “exploratory abuse (diagnostic)” we only selected the cases subjected to celioscopy, strictly relying on this purpose, taking out the procedures in which laparoscopy had curative intentions from the beginning. 

Obviously, due to this selection, many patients’ cases in which the minimally invasive technique completed the pre-operatory diagnosis by the intra operatory exploration period (even though the operation was completed in the pre-established manner), were eliminated from the study.

In cases when after the ultrasound and computer tomography the diagnosis remained uncertain, or when the biopsy was imposed to retrieve biological evidence necessary for the bacteriological exam and histopathology, we considered laparoscopy to be the only convenient way of exploration. 

In case of diagnosis of a complex pathology, with important inflammatory remedies or complex congenital malformations, the celioscopic procedure can be resumed to the exploratory period, which has lower risks in comparison to a therapeutic act, even if it is minimally invasive. On the other hand, the necessary conversions that appear during therapeutic laparoscopies, reflect in fact intra operatory incidents and accidents, and give us a realistic overview on the celioscopic treatment limits. In time, gathering experience, the pediatric surgeon with laparoscopic abilities, is able to make the difference between a major intra-operatory incident and a minor one.

Esposito and Porreca from the University Federico II in Naples, describe a case of hemoretroperitoneum that appeared during an exploratory laparoscopy in a 5 year old patient, that presented a severe neurological handicap (scoliosis and gastro- esophageal reflux). The bleeding source appeared to be a lesion of the vascular iliac vessels that lead to a major xiphoid –pubic incision, to control the hemorrhage [**5**].

According to a study performed in 1994, in many centers in France, vascular complications during laparoscopic pediatric surgery are rare (0, 3-3%).

Analyzing two study groups (exploratory and therapeutic) we establish the superiority of intra operatory diagnosis elements obtained when performing an exploratory laparoscopy, in comparison with elements observed during a therapeutic celioscopy. In Table V, we present some elements of intra-operatory diagnosis for each study group. We can observe that although celioscopy itself represents a source of surprises, these “small” surprises can be possible when the surgeon is confident in the pre-operatory diagnosis and can change the therapeutic tactic. 

**Table V:** Intra operatory laparoscopic diagnosis elements

*Diagnosis elements*	*Therapeutic laparoscopies (% of 580 cases)*	*Exploratory laparoscopies (% of 83 cases)*
Diffused peritonitis	2 (0, 34%)	5 (6, 02%)
Hemoperitoneum	1 (0, 17%)	4 (4, 81%)
Adherence syndrome	2 (0, 34%)	4(4, 81%)
Inflammatory genital syndrome	2 (0, 34%)	4 (4, 81%)
Ectopic testis	1(0, 17%)	14 (16, 86%)
Congenital malformation	1 (0, 17%)	9 (10, 84%)
TOTAL	9 (1, 53%)	40 (48, 15%)

**Fig. 2:**
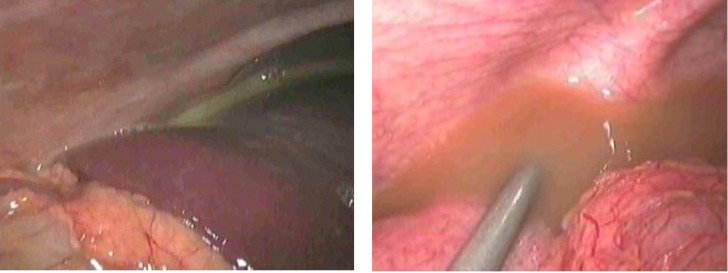
Diffused purulent peritonitis

**Fig. 3:**
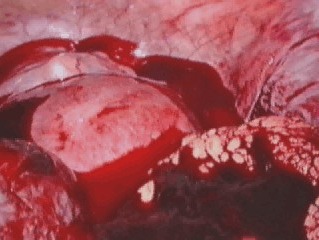
Hemoperitoneum

**Fig. 4:**
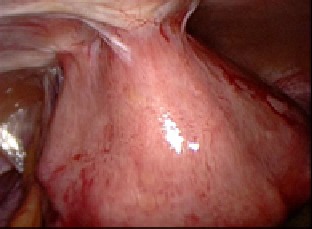
Adherence syndromes

**Fig. 5:**
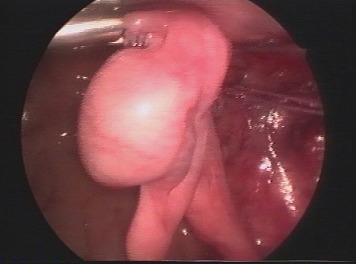
Ectopic testis

**Fig. 6:**
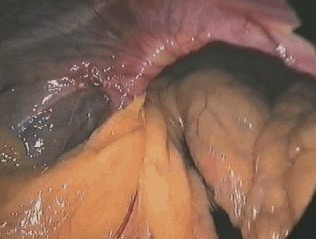
Congenital malformation- Diaphragmatic hernia

All these diagnosis elements, which the surgeon presumed from the beginning, have been omitted in some major or minor manner by other clinical exams (ultrasound, computer tomography). As an example, ultrasound is able to detect 94% of the intra abdominal testis [**12**]. Laparoscopy proved to be superior to ultrasound, computer tomography and magnetic resonance, in many situations [**13**].

SAGES (Society of American Gastrointestinal Endoscopic Surgeons) recommend as methods of pre operatory investigations: abdominal X ray, ultrasound, computer tomography, nuclear magnetic resonance [**14**].

## Conclusions 

• The method has to be considered as a convenient alternative to other diagnosis procedures, offering the great advantage of a possible therapeutic act to follow.

• In many cases, celioscopy is followed by therapeutic attempts: minimally invasive, classic and medical. 

• The difference is well known between the important surgical trauma (major, most of the times) and diagnostic benefits (sometimes minor) conferred by open surgery in pediatric patients.

• The procedure gathers extra diagnostic elements compared to non-invasive methods. Exploratory laparoscopy represents a complementary diagnostic procedure, a laborious one, but minimally invasive.

• The therapeutically correct conduct is represented with the help of the surgical decisional management algorithm of the pediatric patient and the exploratory laparoscopic indication algorithm in pediatric pathology.
